# Case report: A variant of the *FIG4* gene with rapidly progressive amyotrophic lateral sclerosis

**DOI:** 10.3389/fneur.2022.984866

**Published:** 2022-08-24

**Authors:** Mubalake Yilihamu, Xiaolu Liu, Xiaoxuan Liu, Yong Chen, Dongsheng Fan

**Affiliations:** ^1^Department of Neurology, Peking University Third Hospital, Beijing, China; ^2^Beijing Municipal Key Laboratory of Biomarker and Translational Research in Neurodegenerative Diseases, Beijing, China; ^3^Key Laboratory for Neuroscience, National Health Commission/Ministry of Education, Peking University, Beijing, China

**Keywords:** amyotrophic lateral sclerosis, FIG4, mutation, genetics, case report

## Abstract

Heterozygous autosomal-dominant *FIG4* mutations are associated with amyotrophic lateral sclerosis (ALS). Here, we describe a variant of the *FIG4* gene (c.350dupC, p.Asp118GlyfsTer9) in a patient with rapidly progressive ALS that has not previously been reported in ALS or primary lateral sclerosis (PLS) patients before. Our study provides further information on the genotypes and phenotypes of patients with *FIG4* mutations.

## Introduction

Amyotrophic lateral sclerosis (ALS) is a devastating neurodegenerative disease that selectively impairs the motor cortex, the motor neurons of the brainstem and the spinal cord (1). Nearly 10% of ALS cases are classified as familial ALS (FALS), whereas the remaining 90% of cases are considered sporadic ALS (SALS) (2). Factor-induced gene 4 (*FIG4*), also known as *SAC3*, encodes a phosphatase that regulates phosphatidylinositol 3,5-bisphosphate, a molecule critical for intracellular vesicle trafficking along the endosomal-lysosomal pathway (3). Mutations of *FIG4* lead to the development of Charcot-Marie-Tooth disease type 4J (CMT-4J), ALS and primary lateral sclerosis (PLS) (4). Until now, only a few clinical reports of patients with ALS with *FIG4* mutations exist (5). We report the case of a 55-year-old Chinese patient with rapidly progressing ALS possibly associated with a heterozygous *FIG4* mutation (c.350dupC, p.Asp118GlyfsTer9). To date, this mutation has not been described in ALS patients.

## Case report

A 55-year-old man without a personal or familial history of neuromuscular disease started to experience progressive muscle weakness and atrophy in the upper and lower limbs and then rapidly developed dysarthria and dysphagia. Due to respiratory failure, the patient underwent tracheostomy only 10 months after symptom onset. He now communicates with the outside world mostly using eye movement technology. The diagnostic delay was 4 months, and the follow-up examinations did not show any cognitive impairment. Neurological examination revealed non-ambulatory tetraparesis with hyperactive deep tendon reflexes, tongue atrophy and positive Hoffman and Babinski signs. Sensory and cerebellar functions were normal. According to the El Escorial revised criteria (6), the diagnosis of definite ALS was made. Brain and spinal cord magnetic resonance imaging (MRI) were normal. Electromyography (EMG) showed some fibrillation potentials and fasciculation in the sternocleidomastoid, the first dorsal interosseous, the rectus abdominis and the tibialis anterior muscles. The results of routine blood analyses and tests for infections, cancer, autoimmune diseases, vitamin deficiencies, and toxic/metabolic diseases were all normal or negative. The findings of cerebrospinal fluid tests were unremarkable.

**Figure 1:**
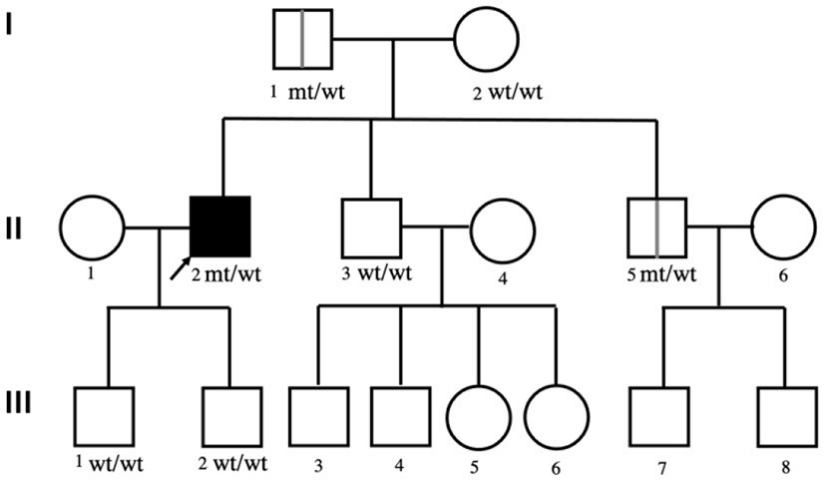
The pedigree of the affected family. The squares indicate males; the circles indicate females; the black symbols indicate affected individuals; the arrow indicates the proband; wt/wt indicates homozygous wild-type.

The affected family pedigree is shown in Figure 1. The patient's parents, brothers, and his two sons are all living and maintain good health, with no family history of neurological diseases. His mother (I-2), one of his brothers (II-3) and his two sons (III-1 and III-2) were healthy, and no positive neurological signs were identified. While his father (I-1) and his other brother (II-2) were clinically asymptomatic, physical examinations showed some positive signs. At 81 years of age, physical examination revealed that the patient's father (I-1) had grade V muscle strength; normal muscle tone; and positive palmomental reflex, suck reflex and Babinski signs. The father did not undergo an EMG test. At 50 years of age, physical examination revealed that the patient's brother (II-2) had grade V muscle strength; normal muscle tone; hyperactive deep tendon reflexes; and positive suck reflex and Hoffman, Rossolimo and Babinski signs. The EMG results for brother (II-2) were normal.

**Figure 2:**
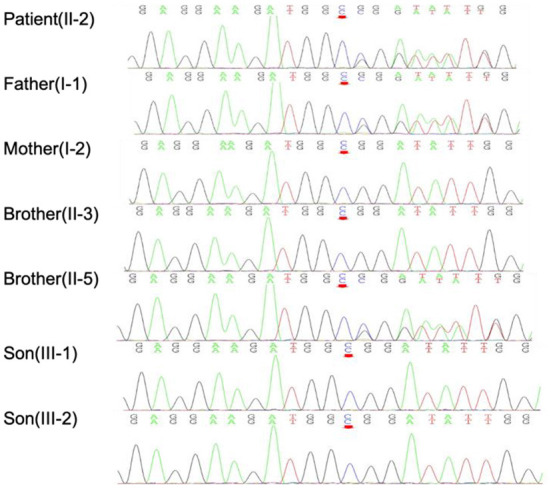
Sanger verification results indicated that the patient and his family members harbored the *FIG4* mutation c.350dupC, as indicated by the red arrow.

First, *PMP22, SOD1, TARDBP, FUS, C9orf72, KIF5A*, and *DCTN1* genetic screenings were performed, yielding negative results. Whole-exome sequencing (WES) was then performed on the patient. Data were analyzed aligned to the human reference genome GRCh37 using Burrows–Wheeler Aligner (BWA), Samtools and Picard, while variant calls were obtained using GATK. Variants were filtered for an allele frequency of <0.01 according to the following online databases: the Short Genetic Variations Database (dbSNP) (https://www.ncbi.nlm.nih.gov/snp), the 1000 Genomes Project (1000G) database (http://www.1000genomes.org/), the Exome Aggregation Consortium (ExAC) database (http://exac.broadinstitute.org/), and gnomAD (http://gnomad.broadinstitute.org/). Sanger validation identified a heterozygous mutation, c.350dupC:p.Asp118GlyfsTer9, of *FIG4* in this patient, his father (I-1) and his brother (II-2) that was not present in his other family members (Figure 2). No suspicious copy number variations (CNVs) were identified in the individuals carrying the *FIG4* variant. This frameshift mutation, NM_014845: exon 4: c.350dupC: p.Asp118GlyfsTer9 (HGVS name), which has not been previously recorded in gene mutation databases, including gnomAD, ExAC, the China Metabolic Analytics Project (ChinaMAP, www.mBiobank.com), and the online Chinese Millionome Database (CMDB, https://db.cngb.org/cmdb/), was identified as a novel mutation. We predicted the impact of this variant on protein function through the use of PolyPhen-2 (http://genetics.bwh.harvard.edu/pph2/), MutationTaster software (http://www.mutationtaster.org) and Combined Annotation Dependent Depletion (CADD, https://cadd.gs.washington.edu/), but no results were obtained. According to the American College of Medical Genetics and Genomics (ACMG) standards and guidelines, the variant was interpreted as likely pathogenic of ALS (PVS1, PM2) since it leads to a null allele and possibly a deleterious effect (PVS1) and is not present in the control databases (gnomAD, ExAC, or dbSNP) (PM2).

## Discussion

Heterozygous autosomal-dominant *FIG4* mutations have been associated with ALS, specifically ALS11 (7). Most of the studies on *FIG4* variants and ALS found that the ALS patients carrying *FIG4* variants are more likely to progress slowly. Some researchers found that the European ALS patients carrying *FIG4* variants were significantly upper motor neuron (UMN) predominant, and the disease duration of these patients was longer. These researchers proposed that if identified in ALS patients, *FIG4* variants may serve as markers for a relatively good prognosis (5). Another Caucasian ALS patient presented with slowly progressing motor neuron disease and frontotemporal dementia (FTD), possibly associated with a heterozygous *FIG4* mutation (8). Some Chinese researchers have also identified *FIG4* variants in Chinese ALS patients. Zhang et al. (9) identified two *FIG4* missense variants (c.658A>G p.Ile220Val and c.2063A>G p.Asp688Gly) of uncertain significance in Chinese SALS patients without a long disease duration. Recently, Chinese researchers Liu et al. (10) used targeted next-generation sequencing to identify novel *FIG4* variants (c.352G>T, p.Asp118Tyr and c.2158G>T, p.Glu720^*^) in two Chinese SALS patients. The patient carrying the *FIG4* p.D118Y variant also presented with progressive ALS, with a Revised Amyotrophic Lateral Sclerosis Functional Rating Scale (ALSFRS-R) score decreasing by 0.4 per month, although this patient still showed milder progression than our patient. One Italian ALS patient with a *FIG4* variant was reported to have a disease onset at a very young age, with a rapid disease course, but the patient also had relevant cognitive impairment (11).

We identified a novel c.350dupC, p.Asp118GlyfsTer9 mutation located in exon 4 of the *FIG4* gene that is possibly associated with ALS. This variant thus far has not been described in patients with ALS, PLS, or CMT. Our patient with ALS presented an atypical phenotype compared with that of previously reported *FIG4*-variant-related cases. The most distinguishing feature was the very rapid disease course without cognitive impairment, reaching clinical endpoints only 10 months after symptom onset. Bertolin et al. (11) also reported the case of a patient with rapidly progressive ALS, a 25-year-old female carrying the *FIG4* variants c.1667C>T p.T556I and c.122T>C. p.I41T together; however, our patient carried only one variant.

**Figure 3:**
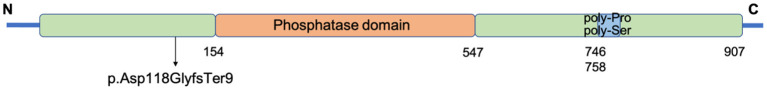
Schematic graph of the *FIG4* protein.

We summarize all frameshift variants and non-sense variants of the *FIG4* gene in ALS patients that have been reported thus far in Table 1. Most of the disease durations of these patients are not long, and some of them also carry other ALS variants. We found that patients with variants that were closer to the C-terminus had longer disease courses. The *FIG4* protein is composed of three domains, an interaction domain at the N-terminus, a phosphatase domain in the central region, and poly-Pro and poly-Ser domains at its C-terminus (13). In this case, the patient carried the p.Asp118GlyfsTer9 variant, which is near the N-terminal (as shown in Figure 3), is predicted to produce a shortened protein without the phosphatase domain and also the catalytic domain, including the active center P-loop. Therefore, the rapid progression of our patient is quite distinctive. The first two very rapidly progressing variants shown in Table 1 (p.R23fs^*^30 and p.D118Gfs^*^9) both result in the loss of expression of *FIG4* protein at the mRNA level, which is important in intracellular vesicle trafficking along the endosomal-lysosomal pathway. Further biological studies investigating mutations in different domains of the *FIG4* gene are still needed.

**Table 1:** Clinical phenotypes of frameshift variants and non-sense variants of the *FIG4* gene identified in ALS patients.

**References**	**c.DNA**	**Variant**	**Exon**	**Phenotype**	**Sex**	**Age of onset**	**Disease duration (yr)**
Chow et al. (7)	c.67-1G>T	p.R23fs*30	2	FALS	Male	77	1.3
This study	c.350dupC	p.D118Gfs*9	4	SALS	Male	55	0.83
Chow et al. (7)	c.547C>T	p.R183X	6	SALS	Male	62	8.9
Osmanovic et al. (5)	c.759delG	p.F254Sfs*8	7	FALS	Male	40	2.67^+^
Lamp et al. (12)	Not available	p.I345Yfs*17 (co-occurrence of C9orf72 repeat expansions)	9	FALS	Female	65	3
Chow et al. (7)	c.1207C>T	p.Q403X	11	SALS	Female	60	25
Liu et al. (10)	c.2158G>T	p.E720X	19	SALS	Male	62	11.5

^+^indicates that individuals are still alive.

The patient's father and brother, who were clinically asymptomatic, carried the c.350dupC mutation, and physical examinations showed some positive signs. We make two assumptions. First, we hypothesize that the variant may be incompletely penetrant for the following reasons. Some researchers have identified incomplete penetrance of a variant in a European family with ALS, which includes an unaffected father carrying a *FIG4* frameshift variant, c.759delG, p.(F254Sfs^*^8), suggesting that *FIG4* variants are not causative alone but that rare variants in multiple genes may need to be carried for individuals to show disease (5). This notion is corroborated by the finding that at least 30 genes are implicated in ALS pathogenesis, with a low percentage of ALS cases being explained by variants in each of these genes (14). Therefore, we speculate that our patient may also carry other ALS pathogenic variants with a low mutation rate that caused his ALS, which we did not detect, while his father and brother may not carry these variants. Second, the patient's father and brother may have had PLS, which would mean that the same gene mutation in a family manifested different diseases. Continual follow-up of these two family members is needed, as well as further functional verification.

## Data availability statement

The datasets presented in this article are not readily available because of ethical and privacy restrictions. Requests to access the datasets should be directed to the corresponding author/s.

## Ethics statement

The studies involving human participants were reviewed and approved by the Ethics Committees of Peking University Third Hospital (Beijing, China). The patients/participants provided their written informed consent to participate in this study. Written informed consent was obtained from the individual(s) for the publication of any potentially identifiable images or data included in this article.

## Author contributions

MY collected the data and wrote and submitted the manuscript for publication. XiaolL and XiaoxL diagnosed and treated the patient. YC and XiaolL revised the manuscript. DF followed the family members and reviewed and edited the manuscript. All authors contributed to the article and approved the submitted version.

## Funding

This work was funded by the National Natural Science Foundation of China (Grant 81873784 and 8207142).

## Conflict of interest

The authors declare that the research was conducted in the absence of any commercial or financial relationships that could be construed as a potential conflict of interest.

## Publisher's note

All claims expressed in this article are solely those of the authors and do not necessarily represent those of their affiliated organizations, or those of the publisher, the editors and the reviewers. Any product that may be evaluated in this article, or claim that may be made by its manufacturer, is not guaranteed or endorsed by the publisher.
